# Dental Follicle Cells Rescue the Regenerative Capacity of Periodontal Ligament Stem Cells in an Inflammatory Microenvironment

**DOI:** 10.1371/journal.pone.0108752

**Published:** 2014-10-02

**Authors:** Jia Liu, Liying Wang, Wenjia Liu, Qiang Li, Zuolin Jin, Yan Jin

**Affiliations:** 1 State Key Laboratory of Military Stomatology, Department of Orthodontics, School of Stomatology, The Fourth Military Medical University, Xi’an, Shaanxi, China; 2 State Key Laboratory of Military Stomatology, Center for Tissue Engineering, School of Stomatology, The Fourth Military Medical University, Xi’an, Shaanxi, China; 3 Research and Development Center for Tissue Engineering, Fourth Military Medical University, Xi’an, Shaanxi, China; 4 State Key Laboratory of Military Stomatology, Department of General Dentistry & Emergency, School of Stomatology, The Fourth Military Medical University, Xi’an, Shaanxi, China; Instituto Butantan, Brazil

## Abstract

**Aims:**

Periodontal ligament stem cells (PDLSCs) are one of the best candidates for periodontal regeneration. Their function could be impaired in periodontitis microenvironment. Dental follicle cells (DFCs), serving as precursor cells and mesenchymal stem cells, have intimate connection with PDLSCs. However, it is still unknown whether DFCs could provide a favorable microenvironment to improve the proliferation and differentiation capacity of PDLSCs from healthy subjects (HPDLSCs) and patients diagnosed with periodontitis (PPDLSCs).

**Methods:**

HPDLSCs, PPDLSCs and DFCs were harvested and identified using microscopic and flow cytometric analysis. Then, the coculture systems of DFCs/HPDLSCs and DFCs/PPDLSCs were established with 0.4 µm transwell, in which all the detection indexs were obtained from HPDLSCs and PPDLSCs. The expression of stemness-associated genes was detected by real-time PCR, and the proliferation ability was assessed using colony formation and cell cycle assays. The osteogenic differentiation capacity was evaluated by real-time PCR, western blot, ALP activity, Alizarin Red S staining and calcium level analysis, while the adipogenic differentiation capacity was determined by real-time PCR and Oil Red O staining. The cell sheet formation in vitro was observed by HE staining and SEM, and the implantation effect in vivo was evaluated using HE staining and Masson’s trichrome staining.

**Results:**

PPDLSCs had a greater proliferation capability but lower osteogenic and adipogenic potential than HPDLSCs. DFCs enhanced the proliferation and osteogenic/adipogenic differentiation of HPDLSCs and PPDLSCs to different degrees. Moreover, coculture with DFCs increased cell layers and extracellular matrix of HPDLSCs/PPDLSCs cell sheets in vitro and improved periodontal regeneration by HPDLSCs/PPDLSCs in vivo.

**Conclusions:**

Our data suggest that the function of PPDLSCs could be damaged in the periodontitis microenvironment. DFCs appear to enhance the self-renewal and multi-differentiation capacity of both HPDLSCs and PPDLSCs, which indicates that DFCs could provide a beneficial microenvironment for periodontal regeneration using PDLSCs.

## Introduction

Periodontitis is a chronic infectious disease that can lead to the destruction of periodontal tissues and even tooth loss [1], [2]. Therapeutic strategies for the treatment of periodontitis include not only the control of local inflammation but also the regeneration of new periodontal tissues attached to the surface of the tooth root. Although stem cell biology and guided tissue regeneration (GTR) have provided advances in inflammation control, they still have limitations for the recovery of a functional periodontium [3].

Tissue engineering has recently been shown to be a promising approach for periodontal regeneration [4]–[6], and strategies using mesenchymal stem cells (MSCs) are especially promising [7]. Periodontal ligament stem cells (PDLSCs) have been identified as a type of MSCs present in periodontal tissues and are capable of differentiating into cementum-forming cells, bone-forming cells, adipocytes and collagen-forming cells. After transplantation into immunocompromised mice, PDLSCs are able to generate cementum/PDL-like structures [8]–[11]. Compared with MSCs from other tissue sources, PDLSCs are more similar to the native periodontal tissues with regard to morphology, structure and characteristics, making them the best candidate for periodontal regeneration [12]–[14]. Therefore, optimizing the characteristics and function of PDLSCs to regenerate periodontal tissues (including fibrous tissues and bones) is an important topic in this field.

The extracellular microenvironment is known to affect the proliferation and differentiation of MSCs [15]–[17]. It has previously been demonstrated that the periodontitic microenvironment can decrease the osteogenic ability of PDLSCs [18]. In contrast, a favorable microenvironment, such as that provided by conditioned medium from young periodontal ligament cells, can enhance the proliferation and differentiation of PDLSCs from aged donors [19].

Dental follicle cells (DFCs), which are a type of MSCs found in periodontal tissues, are young precursor cells present during tooth development [20]. DFCs are intimately associated with PDLSCs, both structurally and functionally, during tooth development. In this study, we established a co-culture system for DFCs and PDLSCs using transwell to simulate the natural microenvironment present during tooth development. PDLSCs were obtained from healthy subjects (HPDLSCs) and patients diagnosed with periodontitis (PPDLSCs). We postulated that DFCs, as a homologous precursor cell type, could provide a beneficial microenvironment to optimize the characteristics of PDLSCs (both HPDLSCs and PPDLSCs) through cell-to-cell interactions.

## Materials and Methods

### Study Subjects and Ethics Statement

HPDLSCs for primary culture (n = 15) were obtained from healthy periodontal tissues of 10 orthodontic patients (25–43 years old) undergoing premolar and third molar extractions. PPDLSCs for primary culture (n = 10) were obtained from 6 periodontitis patients (28–52 years old) who were diagnosed by the same periodontics specialist based on the clinical manifestation of alveolar bone loss (≥1/3) and more than one periodontal pocket (depth≥5 mm). DFCs for primary culture (n = 8) were obtained by culturing tissue explants from healthy subjects whose third molars were being extracted for orthodontic reasons during the phase of tooth germ development. The subjects included in this study did not have a history of systemic disease, smoking or special medication. All samples were collected at the Department of Oral and Maxillofacial Surgery of the School of Stomatology at the Fourth Military Medical University. All participants provided written informed consent, and the study was approved by the Ethics Committee of School of Stomatology, Fourth Military Medical University (Xi’an, China).

### Cell Culture

For cell isolation, the teeth were first washed in sterile phosphate-buffered saline (PBS). The periodontal ligament (PDL) was then gently separated from the middle part of the root surface and cut into small pieces (1 mm^3^) under a microscope. Colonies were established from single cells using the limiting dilution technique to obtain homogeneous populations of HPDLSCs and PPDLSCs, as previously described [21], [22]. The culture medium was changed every 2 days. After 2–3 weeks of culture, the single cell-derived clones were harvested and mixed together. Multiple colony-derived HPDLSCs and PPDLSCs at passage 2–4 were used in our experiments. For each experiment, PDLSCs at the same passage were used.

### Flow Cytometric Analysis

For identification of the MSC phenotype, approximately 5×10^5^ PDLSCs were incubated with PE-conjugated monoclonal antibodies against human Stro-1, CD29, CD45, CD90, CD105 and CD146 (BD Bioscience, San Jose, CA, USA). The cells were incubated with the specified antibodies for 1 hour in a 4°C lucifugal environment. After washing three times with PBS containing 30 ml/L fetal bovine serum (FBS), the cells were subjected to flow cytometric analysis using a Beckman CoulterEpics XL instrument (Beckman Coulter, Fullerton, CA, USA) for cell phenotype characterization.

For cell cycle analysis, HPDLSCs and PPDLSCs (approximately 2×10^4^ cells) were cultured in 6-well plates with serum-free α-MEM, and DFCs (5×10^4^) were seeded in transwell chambers with 0.4 µm pores (Millicell, Millipore, MA, USA). After culturing for 24 hours, the transwell was placed into the 6-well plates to establish the DFC/HPDLSC and DFC/PPDLSC co-culture systems. The control groups consisted of monocultured HPDLSCs or PPDLSCs in the plates, with no cells in the transwell chambers. The monocultured and co-cultured HPDLSCs and PPDLSCs were maintained separately in their own systems for 5 days. Then, single-cell suspensions (5×10^5^ HPDLSCs and PPDLSCs each) were washed with PBS containing 30 ml/L FBS, fixed with 75% ethanol and subjected to cell cycle analysis by flow cytometry. The fractions of cells in the G_1_, S and G_2_ phases of the cell cycle were measured, and the proliferation index (PI, the percentage of cells in G_2_+S phases) was analyzed.

### Colony-Forming Assays

Single-cell suspensions (50 cells) of HPDLSCs and PPDLSCs in α-MEM (10% FBS) were seeded in 6-well plates (Corning, Lowell, MA, USA), and DFCs (5×10^4^) were seeded in the transwell chambers (Millipore, Billerica, MA, USA). After 6 hours, the co-culture and monoculture systems were established, as described above. The HPDLSCs and PPDLSCs cultures were fixed in 4% paraformaldehyde and stained with 0.1% toluidine blue at day 10. The stained cultures were analyzed by microscopy, and aggregates containing 50 or more cells were counted as colonies. The number of colonies per well was counted, and the colony-forming rate was calculated.

### Real-time PCR

Total RNA was isolated from the HPDLSCs and PPDLSCs using Trizol reagent (Invitrogen, Carlsbad, CA, USA), and reverse transcription was performed using the PrimeScript RT reagent kit (Takara, Bio, Otsu, Japan). The primer sequences used in the experiment are listed in Table 1. Real-time PCR reactions were performed using the SYBR Premix Ex Taq II kit (Takara, Bio, Otsu, Japan) and an applied Bio-systems CFX96TM Real-Time sequence detection system (Applied Biosystems, Darmstadt, Germany).

**Table 1:** Primer sequences.

Gene	Primer sequence
β-actin	Forward 5′-TGG CAC CCA GCA CAA TGA A-3′
	Reverse 5′-CTA AGT CAT AGT CCG CCT AGA AGC A-3′
Klf4	Forward 5′-GAG CCC AAG CCA AAG AGG-3′
	Reverse 5′-ATC CAC AGC CGT CCC AGT C-3′
Sox2	Forward 5′-ATG GGT TCG GTG GTC AAC TC-3′
	Reverse 5′-CGC TCT GGT AGT GCT GGG A-3′
Oct4	Forward 5′-CCT GTC TCC GTC ACC ACT CTG-3′
	Reverse 5′-AAC CCT GGC ACA AAC TCC-3′
Runx2	Forward 5′-CCC GTG GCC TTC AAG GT-3′
	Reverse 5′-CGT TAC CCG CCA TGA CAG TA-3′
ALP	Forward 5′-GGA CCA TTC CCA CGT CTT CAC-3′
	Reverse 5′-CCT TGT AGC CAG GCC CAT TG-3′
OCN	Forward 5′-CCC AGG CGC TAC CTG TAT CAA-3′
	Reverse 5′-GGT CAG CCA ACT CGT CAC AGT C-3′
PPARγ	Forward 5′-CCA CTT TGA TTG CAC TTT GGT ACT CTT G-3′
	Reverse 5′-CTT CAC TAC TGT TGA CTT CTC CAG CAT TTC-3′

### Osteogenic and Adipogenic Differentiation

For osteogenesis, HPDLSCs and PPDLSCs were plated at a density of 5×10^4^ cells per well in 6-well plates, and DFCs were plated in transwell chambers (Millipore, Billerica, MA, USA) at the same density to establish co-culture and monoculture systems. After reaching 80% confluence, HPDLSCs and PPDLSCs were cultured in osteogenic medium (α-MEM supplemented with 5% FBS, 100 nM dexamethasone (Sigma, Santa Clara, CA, USA), 50 pg/ml ascorbic acid (Sigma, Santa Clara, CA, USA) and 5 mM β-glycerophosphate (Sigma, Santa Clara, CA, USA)) for 7–28 days. The media were changed every 3 days. For the differentiation analysis, the cells were washed twice in PBS and fixed in 4% paraformaldehyde for 30 min. Subsequently, alkaline phosphatase (ALP) staining was performed using the BCIP/NBT Alkaline Phosphatase Color Development Kit (Beyotime, Shanghai, China), and ALP activity was measured using the Alkaline Phosphatase (AKP/ALP) Detection Kit (Jiancheng Bioengineering, Nanjing, China). Mineralized nodules were stained with Alizarin Red S (pH 4.2) (Kermel, Tianjin, China) for 15 min at room temperature. Calcium levels were measured using a calcium colorimetric assay kit (BioVision, San Francisco, CA, USA).

For adipogenesis, HPDLSCs and PPDLSCs were plated at a density of 5×10^4^ cells per well in 6-well plates and co-cultured with DFCs at the same density. After reaching 80% confluence, the HPDLSCs/PPDLSCs were cultured in adipogenic medium (α-MEM supplemented with 5% FBS, 0.5 mM methylisobutylxanthine (Sigma, Santa Clara, CA, USA), 0.5 µM hydrocortisone (Sigma, Santa Clara, CA, USA), and 60 µM indomethacin (Sigma, Santa Clara, CA, USA) for 7–21 days. For analysis, the adipogenic cultures were fixed in 4% paraformaldehyde for 30 min and stained with fresh Oil Red O solution (Sigma, Santa Clara, CA, USA) for 15 min. To quantify the amount of Oil Red O-stained lipids, the stain was solubilized in isopropanol for 5 minutes at room temperature. The solubilized stain (150 µL) was then transferred to the wells of a 96-well plate, and the absorbance was measured at 520 nm.

### Protein Isolation and Western Blot Analysis

Total protein was extracted from the cells by lysis in RIPA buffer (10 mM Tris-HCl, 1 mM EDTA, 1% sodium dodecyl sulfate, 1% Nonidet P-40, 1∶100 proteinase inhibitor cocktail, 50 mM b-glycerophosphate and 50 mM sodium fluoride). The protein concentration in the extracted lysates was determined using a protein assay solution based on the absorbance at 595 nm (Bio-Rad, Hercules, CA, USA). Additionally, 20 to 50 mg of the cell lysate samples were separated by 10% SDS-PAGE and then transferred to a polyvinylidene fluoride (PVDF) membrane (Bio-Rad, Hercules, CA, USA). The membranes were blocked with 5% milk for 2 hours and then incubated with anti-Runx2 (Abcam, Cambridge, UK) and anti-β-actin (Cell Signaling Technology, Beverly, MA, USA) primary antibodies. The immune complexes were then incubated with horseradish peroxidase-conjugated anti-rabbit or anti-mouse IgG antibodies (Boshide, Beijing, China). Immunodetection was performed using the Western-Light Chemiluminescent Detection System (Peiqing, Shanghai, China).

### Culture of Cell Sheets

HPDLSCs and PPDLSCs were seeded in 6-well plates at a density of 2×10^5^ cells per well, and DFCs were seeded in transwell chambers at the same density. Co-culture and monoculture systems were set up after 24 hours. After 8–9 days of culture in normal α-MEM medium containing 5% FBS and ascorbate (50 µg/ml), cell-matrix sheets of HPDLSCs and PPDLSCs had formed and could be easily detached from the bottom of the culture plates using a cell scraper.

### H&E Staining

The sheets were fixed in 4% phosphate-buffered paraformaldehyde for 24 h, embedded in paraffin, sectioned longitudinally and stained with hematoxylin and eosin (H&E). The H&E staining results were evaluated using an Olympus BX50 compound microscope (Olympus Optical, Tokyo, Japan).

### Scanning Electron Microscopy (SEM)

To evaluate matrix secretion by the co-cultured and monocultured HPDLSCs and PPDLSCs, specimens were serially dehydrated with 70%, 75%, 80%, 90%, 95%, and 100% alcohol for 5 min each. The specimens were then sputter-coated with gold using a standard protocol and observed by SEM using a scanning electron microscope (Hitachi, Tokyo, Japan).

### Preparation of Ceramic Bovine Bone (CBB) and Chemically Conditioned Root Dentin (CCRD)

Blocks of CBB (Research and Development Center for Tissue Engineering, Fourth Military Medical University, Xi’an, China) [23] were produced from fresh bovine rib bones (Shaanxi Kingbull Slaughterhouse, Xi’an, China) and shaped to a hollow tubule. CCRD specimens were prepared from collected human teeth and remodeled into slices (thickness, 1.0 mm; length, 2.0 mm), then they were treated by a series of chemical procedures as previously described [24].

### Regeneration Effects of HPDLSC and PPDLSC Sheets Combined with CBB and CCRD in Immunodeficient Mice

HPDLSC and PPDLSC cell sheets were prepared and placed on the surfaces of CCRD scaffolds and wrapped from one side to the other. Then, the constructs containing HPDLSC/PPDLSC sheets and CCRD were inserted into CBB tubules to mimic a natural root for heterotopic transplantation into immunodeficient mice. All animal procedures were approved by the Animal Care Committee of the Fourth Military Medical University (Permit Number: 12046), and was conducted according to the Guidelines for Animal Experimentation of the Fourth Military Medical University (Xi’an, China). Six 6-week-old male mice (BALB/c-nu; FMMU Medical Laboratory Animal Center, Xi’an, China) with severe combined immunodeficiency were used as hosts. Each mouse received four samples. Composites of HPDLSC/PPDLSC sheets plus CBB and CCRD scaffolds were transplanted into subcutaneous pockets on the left side; composites of HPDLSCs/PPDLSCs from the monoculture system plus CBB and CCRD scaffolds were implanted on the right side as controls. General anesthesia was administered by intramuscular injection of pentobarbital sodium (0.1 mL/100 g) for all surgical procedures. The mice were checked carefully to prevent infection of the wound, and the health status of animals were observed every day post-transplantation. Eight weeks after implantation, the mice were euthanized by cervical dislocation after general anesthesia, and the implants were removed for H&E and Masson’s trichrome staining.

### Statistical analysis

All experiments were performed in triplicate with three different groups of co-culture systems. All data are expressed as the mean ± standard deviation (S.D.). Statistical significance was assessed with a χ^2^ test and an independent-samples t test using SPSS13.0 software (SPSS, San Rafael, CA, USA). Statistical significance was defined as p<0.05. All data acquisition and analyses were performed in a blinded manner.

## Results

### Culture and Identification of HPDLSCs, PPDLSCs and DFCs

**Figure 1:**
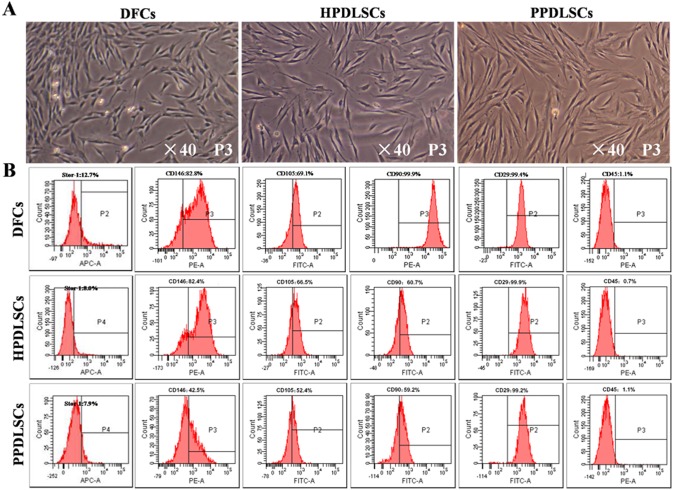
Isolation and identification of HPDLSCs, PPDLSCs, and DFCs. **A:** Morphologies of DFCs, HPDLSCs, and PPDLSCs observed by microscopy. **B:** Mesenchymal stem cell phenotype examination by flow cytometric analysis.

HPDLSCs and PPDLSCs were successfully isolated from PDL tissues derived from healthy donors and patients diagnosed with periodontitis, respectively. Putative stem cells were isolated using a limiting dilution technique and cultured to the third passage. Additionally, DFCs were harvested from dental follicle tissue and cultured to the third passage. Using a microscope, HPDLSCs, PPDLSCs, and DFCs were observed growing in an adherent manner with a long spindle shape. Although no obvious difference was observed between HPDLSCs and PPDLSCs, PPDLSCs appeared slightly irregular (Figure 1A). All three cell populations expressed the mesenchymal stem cell markers Stro-1, CD146, CD105, CD90, and CD29 and were negative for the hematopoietic marker CD45 (Figure 1B). In addition, the experiments described below confirmed the osteogenic and adipogenic potential of the HPDLSCs and PPDLSCs.

### Effect of DFCs on the stemness of HPDLSCs and PPDLSCs

**Figure 2:**
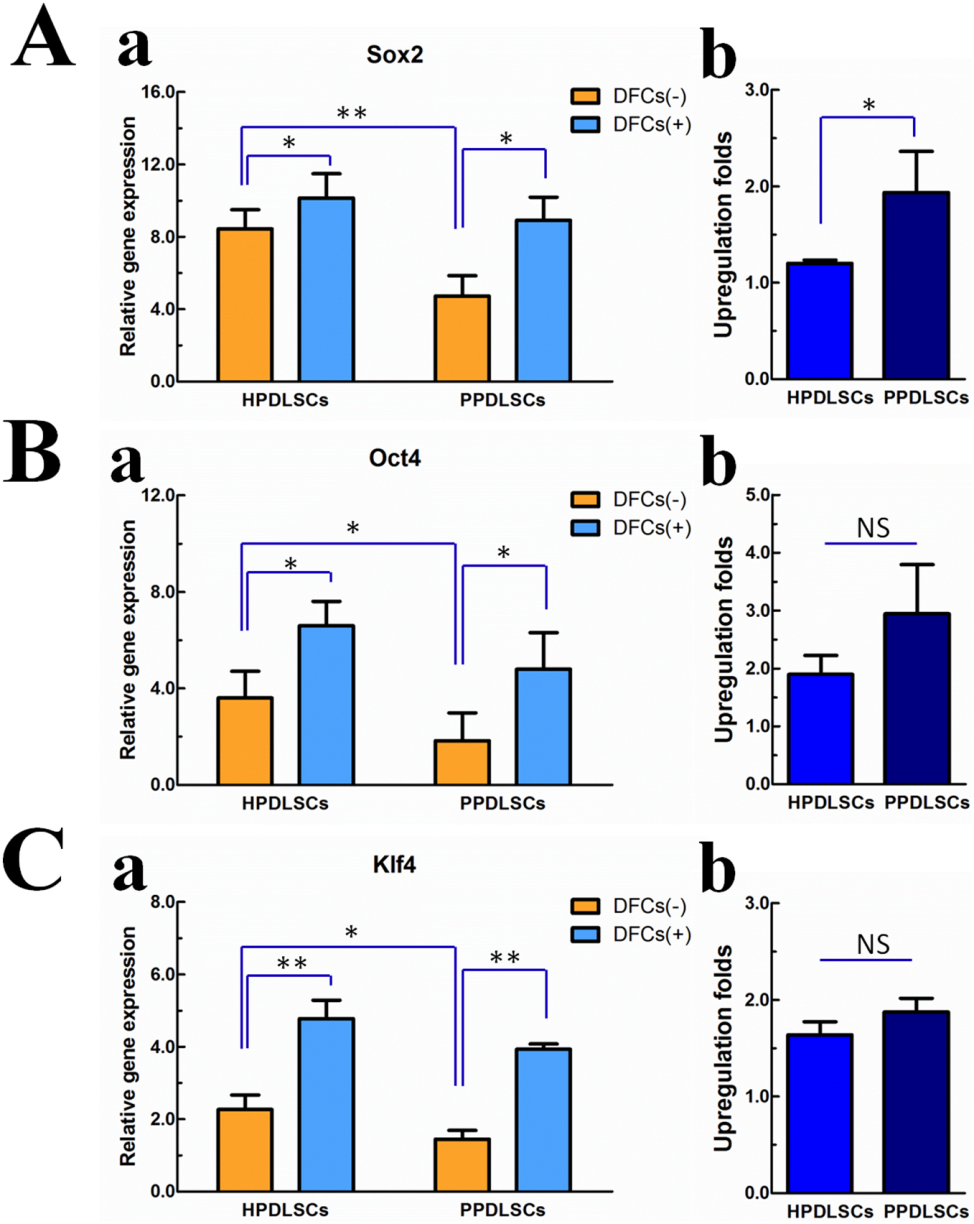
Effects of DFCs on the stemness of HPDLSCs and PPDLSCs. Expression levels of the stemness-related genes Sox2 (**Aa**), Oct4 (**Ba**), and Klf4 (**Ca**) were examined by real time RT-PCR after 14 days of culture with osteogenic supplements. Upregulation folds of Sox2 (**Ab**), Oct4 (**Bb**), and Klf4 (**Cb**) gene levels by coculture with DFCs were quantitatively analyzed in HPDLSCs and PPDLSCs. Notes: DFCs (–), monocultured PDLSCs that were cultured with transwell containing no DFCs; DFCs (+), co-cultured PDLSCs that were cultured with transwell seeded with a specific number of DFCs. Bars represent the mean ± S.D. (n = 3). *p<0.05, **p<0.01, ***p<0.001.

To evaluate the influence of DFCs on the stemness of HPDLSCs and PPDLSCs, real-time PCR was used to evaluate the expression of the stemness-related genes Oct4, Sox2, and Klf4, which are associated with self-renewal and multi-lineage differentiation. PPDLSCs exhibited lower Oct4, Sox2, and Klf4 mRNA expression than HPDLSCs in the monoculture systems; however, in the co-culture systems, DFCs increased the expression of these three genes in both HPDLSCs and PPDLSCs (p<0.05; Figure 2Aa, Ba, Ca). The DFC-mediated upregulation folds of Oct4, Sox2, and Klf4 were higher in PPDLSCs than those in HPDLSCs, especially for Sox2 with statistical significance (p<0.05; Figure 2Ab, Bb, Cb).

### Effect of DFCs on Proliferation of HPDLSCs and PPDLSCs

**Figure 3:**
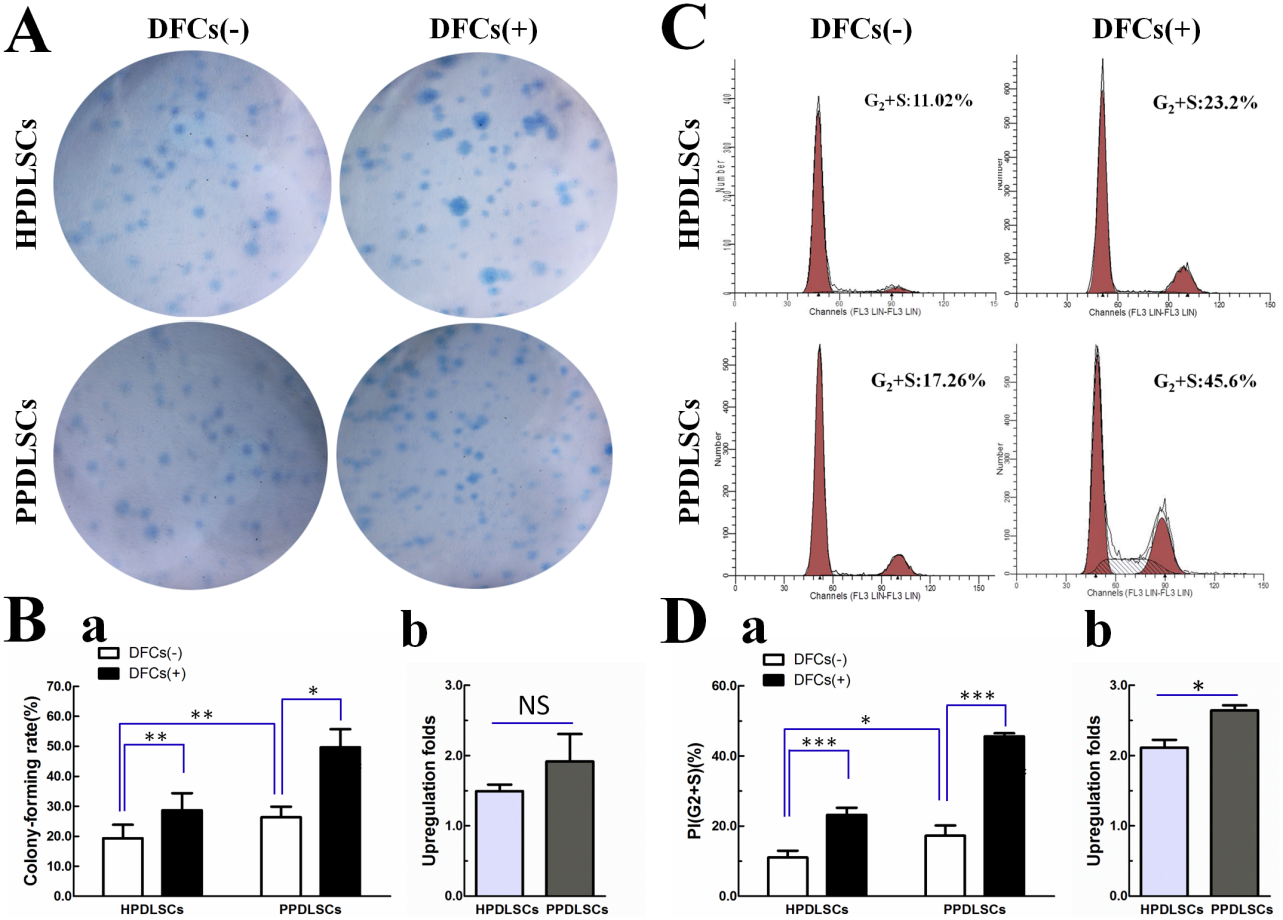
Effects of DFCs on proliferation of HPDLSCs and PPDLSCs. **A:** The colony forming rate was determined using colony-forming assays at day 10. **B:** Quantitative data for the colony-forming assays, **a:** Colony-forming rate of monocultured and cocultured HPDLSCs/PPDLSCs; **b:** Upregulation folds of colony-forming rate by coculture with DFCs in HPDLSCs and PPDLSCs. **C:** The percentage of cells in G2+S phases was measured by flow cytometry at day 5. **D:** Quantitative data of the cell cycle analysis, **a:** PI of HPDLSCs and PPDLSCs in monocultured and cocultured systems; **b:** Upregulation folds of PI in HPDLSCs and PPDLSCs by cocultured with DFCs. Notes: DFCs (–), monocultured PDLSCs that were cultured with transwell containing no DFCs; DFCs (+), co-cultured PDLSCs that were cultured with transwell seeded with a specific number of DFCs. Bars represent the mean ± S.D. (n = 3). *p<0.05, **p<0.01, ***p<0.001.

HPDLSCs and PPDLSCs were cultured in monoculture and co-culture systems, and their proliferation abilities were determined using the colony-forming assay at day 10 and cell cycle analysis at day 5. PPDLSCs had a higher proliferation capacity than HPDLSCs based on their colony-forming rate (p<0.05; Figure 3A, B) and the percentages of cells in G_2_ and S phases (PI) (p<0.05; Figure 3C, D). In addition, co-cultured HPDLSCs/PPDLSCs showed an increased proliferation ability compared to monocultured cells, as indicated by their higher colony-forming rate (p<0.05; Figure 3A, B) and PI (p<0.05; Figure 3C, D). Interestingly, HPDLSCs and PPDLSCs had different sensitivities to DFCs in terms of the observed increase in proliferation. Specifically, DFCs more strongly promoted the proliferation of PPDLSCs than that of HPDLSCs, especially in the cell cycle assay (p<0.05; Figure 3B, D).

### Effects of DFCs on the osteogenic and adipogenic differentiation of HPDLSCs and PPDLSCs in vitro

**Figure 4:**
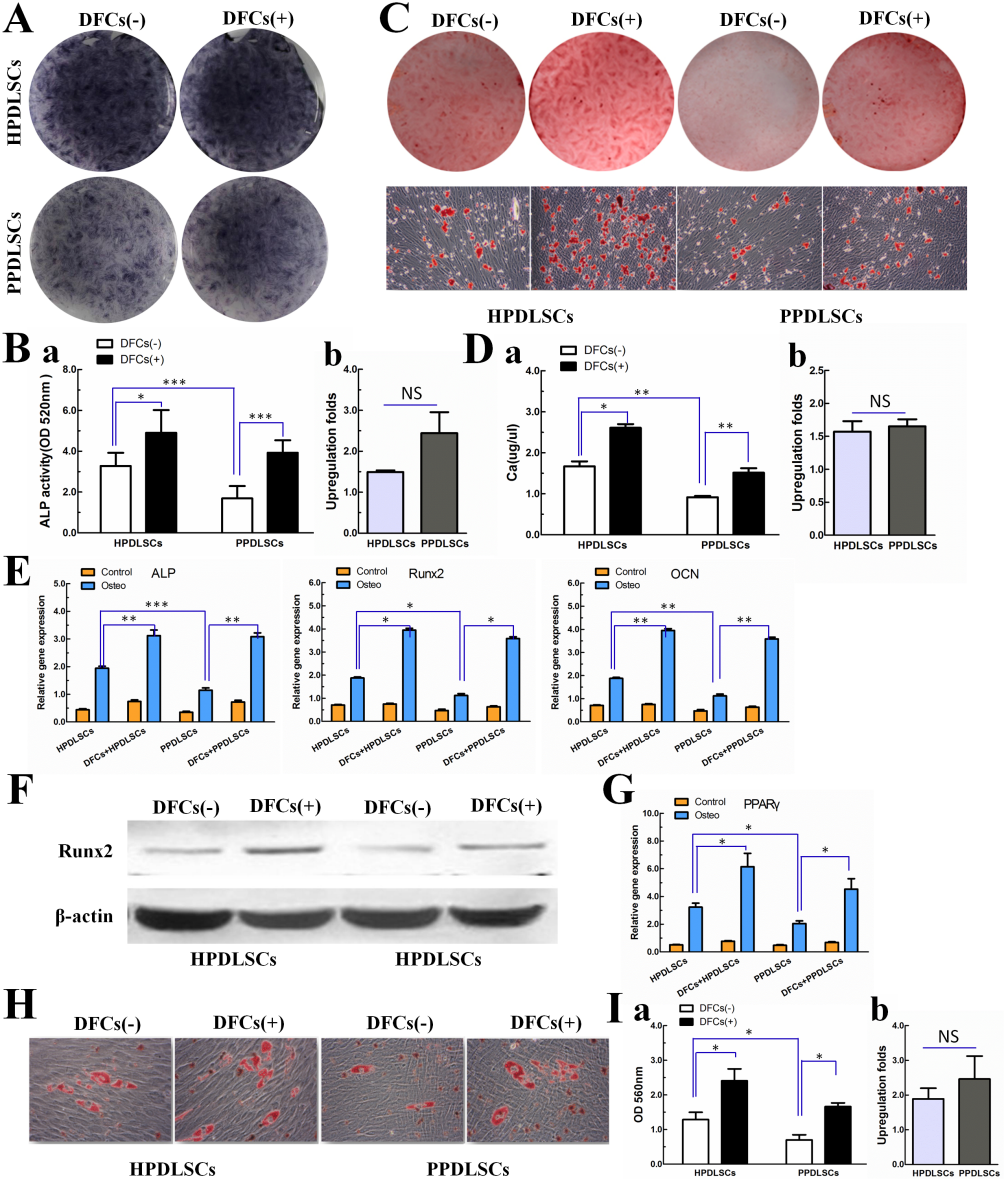
Effects of DFCs on the osteogenic and adipogenic differentiation of HPDLSCs and PPDLSCs in vitro. ALP activity was detected by (**A**) ALP staining and (**Ba**) ALP activity assay at day 7; **Bb:** Upregulation folds of ALP activity in HPDLSCs and PPDLSCs by cocultured with DFCs. **C:** Osteogenic differentiation was determined by Alizarin Red S staining after 28 days of culture with osteogenic supplements. **Da:** Calcium concentration was examined by calcium level analysis after 28 days of culture with osteogenic supplements; **Db**: Upregulation folds of calcium concentration in HPDLSCs and PPDLSCs by cocultured with DFCs. **E:** The expression levels of the osteogenic genes ALP, Runx2, and OCN were measured by real time PCR after 7 days of culture with osteogenic supplements. **F:** Runx2 protein expression was determined by western blot analysis after 7 days of culture with osteogenic supplements. **G:** The expression levels of the adipogenic genes PPARγwas measured by real time RT-PCR after 7 days of culture with adipogenic supplements. **H:** Adipogenic differentiation was evaluated by Oil Red O staining after 21 days of culture with adipogenic supplements. **Ia:** Quantitative data for Oil Red O staining; **Ib:** Upregulation folds of the quantitative data in HPDLSCs and PPDLSCs by cocultured with DFCs. Notes: DFCs (–), monocultured PDLSCs that were cultured with transwell containing no DFCs; DFCs (+), co-cultured PDLSCs that were cultured with transwell seeded with a specific number of DFCs. Bars represent the mean ± S.D. (n = 3). *p<0.05, **p<0.01, ***p<0.001.

At day 7, osteogenic differentiation was analyzed by ALP staining and the ALP activity assay. Additionally, mRNA expression of the osteogenic genes ALP, Runx2, and OCN was determined by real time PCR, and Runx2 protein expression was assessed by western blot analysis. Furthermore, mineralized nodule formation and calcium concentration were examined by Alizarin Red S staining and calcium level analysis at day 28, respectively. HPDLSCs had a better osteogenic capacity than PPDLSCs, with the evidence of higher ALP activity (p<0.05; Figure 4A, Ba), gene expression of ALP, Runx2, and OCN (p<0.05; Figure 4E), protein expression of Runx2 (Figure 4F), as well as more mineralized nodules (Figure 4C) and higher calcium levels (p<0.05; Figure 4Da). These results indicate that the periodontitic microenvironment decreased the osteogenic capacity of PPDLSCs. When we investigated the effect of DFCs on the osteogenic differentiation potential of HPDLSCs and PPDLSCs, we found that DFCs significantly enhanced the osteogenic potential of both HPDLSCs and PPDLSCs in the assays described above (p<0.05; Figure 4A, Ba, C, Da, E, F). Furthermore, co-culture with DFCs appeared to increase the osteogenic potential of PPDLSCs to a greater extent than that of HPDLSCs, based on the quantitative analyses of the upregulation folds of ALP activity (Figure 4Bb) and calcium concentration (Figure 4Db). However, the differences were not statistically significant.

We also evaluated the effects of DFCs on the adipogenic ability of HPDLSCs and PPDLSCs by real time PCR at day 7 and Oil Red O staining at day 21. HPDLSCs showed a higher level of PPARγ gene expression (Figure 4G) and a higher number of lipid droplets than PPDLSCs (p<0.05; Figure 4H, Ia), and co-culture with DFCs increased the adipogenic ability of both HPDLSCs and PPDLSCs (p<0.05; Figure 4H, Ia). Co-culture improved the formation of lipid droplets similarly for PPDLSCs and HPDLSCs (p>0.05; Figure 4Ib).

### Effect of DFCs on cell sheet formation by HPDLSCs and PPDLSCs in vitro

**Figure 5:**
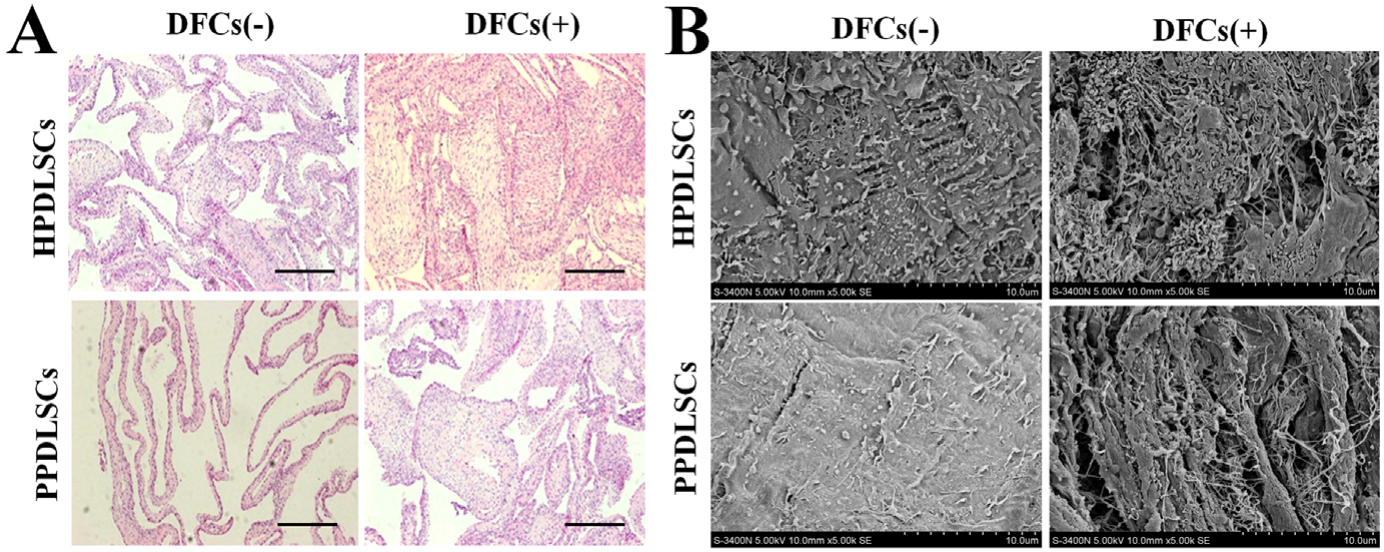
Effects of DFCs on cell sheet formation by HPDLSCs and PPDLSCs in vitro. **A:** H&E staining of cell sheets. HPDLSCs formed more cell layers and ECM than PPDLSCs. In the co-cultured systems, both HPDLSCs and PPDLSCs formed more cell layers and ECM than in the monocultured systems (hematoxylin-eosin staining, magnification: 400×, scale bar = 50 mm). **B:** SEM of cell sheets; HPDLSCs secreted richer ECM than PPDLSCs, and co-culture with DFCs enhanced the ECM secretion by both HPDLSCs and PPDLSCs. Notes: DFCs (–), monocultured PDLSCs that were cultured with transwell containing no DFCs; DFCs (+), co-cultured PDLSCs that were cultured with transwells seeded with a specific number of DFCs.

H&E staining showed that HPDLSCs formed more cell layers and extracellular matrix (ECM) than PPDLSCs in the monoculture groups. Co-culture with DFCs improved cell sheet formation for both HPDLSCs and PPDLSCs. Cell aggregates formed and no necrosis was observed, especially in the co-cultured HPDLSCs (Figure 5A).

SEM showed that in the monoculture groups, HPDLSCs secreted only a limited amount of extracellular matrix (ECM), whereas PPDLSCs only produced a small granule of ECM on the surface of the cell sheets. In the co-culture groups, thick and interwoven ECM was present in both HPDLSC and PPDLSC cell sheets, with more ECM secretion being observed in HPDLSCs (Figure 5B).

### Effect of implantation of cell sheets of HPDLSCs and PPDLSCs in immunodeficient mice

**Figure 6:**
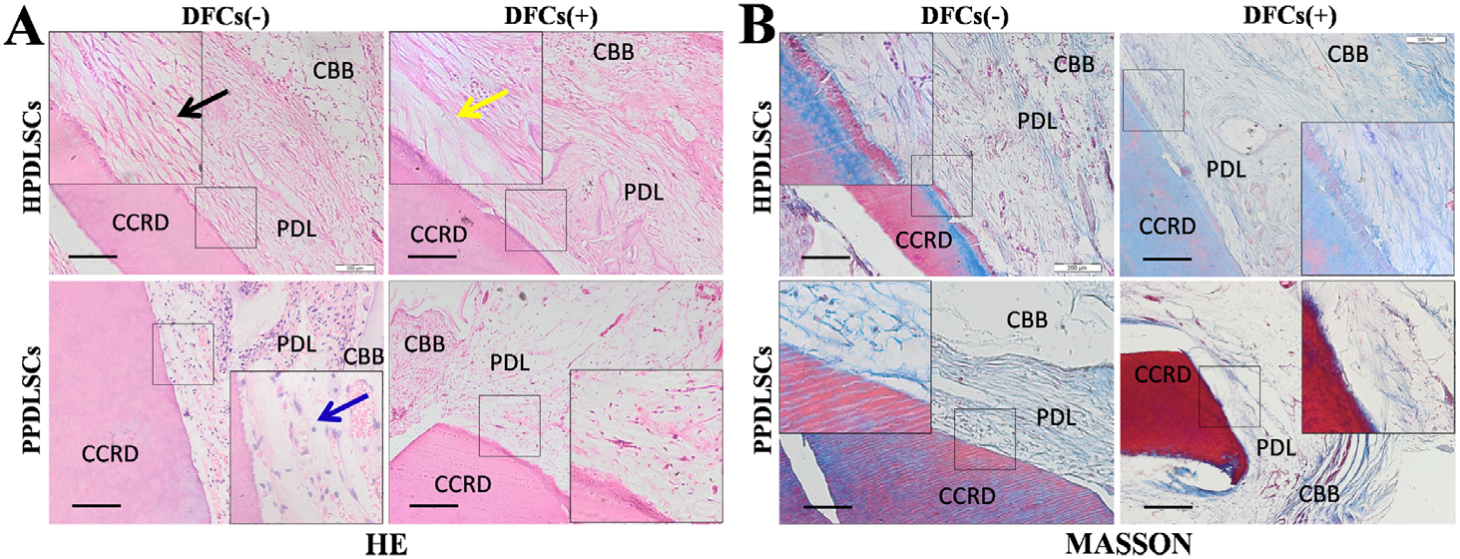
Transplantation of HPDLSC and PPDLSC cell sheets in immunodeficient mice. **A:** H&E staining of cell sheets. Monocultured HPDLSCs formed PDL-like fibers parallel to the CCRD and CBB (black arrow). Co-cultured HPDLSCs formed perpendicular Sharpey-like fibers (yellow arrow) and also generated a root/periodontal ligament-like complex in the CCRD side and periodontal ligament/bone-like complex in the CBB side. In the monocultured PPDLSC group, the fibers did not adhere very well to the CCRD and CBB, and many inflammatory cells were observed in the tissue (blue arrow). In the co-cultured PPDLSC group, no inflammatory cells were observed, and more fibers were formed (hematoxylin-eosin staining, magnification: 400×, scale bar = 50 mm). **B:** Masson’s trichrome staining consistently confirmed the results of the H&E staining for each group (Masson’s trichrome staining: 400×, scale bar = 50 mm). Notes: DFCs (–), monocultured PDLSCs that were cultured with transwell containing no DFCs; DFCs (+), co-cultured PDLSCs that were cultured with transwell seeded with a specific number of DFCs; CBB: ceramic bovine bone; CCRD: chemically conditioned root dentin; PDL: periodontal ligament-like tissue.

After implantation of the cell sheets into the subcutaneous space of immunodeficient mice for 8 weeks, the regenerated tissue specimens were harvested and their morphology was examined by light microscopy after H&E and Masson’s trichrome staining. In the HPDLSC group, several PDL-like fibers were observed in the monocultured HPDLSC sheet, which presented a parallel orientation between the CCRD and CBB. However, in the co-cultured HPDLSC sheet, a typical arranged tissue with Sharpey-like perpendicular fibers was regenerated between the CCRD and CBB. Additionally, a root/periodontal ligament-like complex and a periodontal ligament/bone-like complex were observed on the CCRD and CBB sides, respectively. The collagen fiber bundles between the CCRD and CBB closely resembled the physiological structure of the periodontium. However, in the PPDLSC group, the fibers and bone did not adhere well, and many inflammatory cells were present in the regenerated tissue. In the co-cultured PPDLSC sheet, some Sharpey fiber-like tissue formed between the CCRD and CBB without inflammatory cells (Figure 6A). Masson’s trichrome staining further confirmed the deposition of collagen and the regeneration of mineralized matrix in each group (Figure 6B).

## Discussion

PDLSCs are promising stem cells for periodontal regeneration, as they have been shown to form PDL/cementum and bone-like tissues in vivo [8]–[11]. However, the extracellular microenvironment has a direct effect on PDLSC function [15], [16]. In our study, PDLSCs from two distinct microenvironments were evaluated, i.e. a healthy periodontal environment and an inflammatory periodontal environment. We found that PPDLSCs exhibit high proliferation ability, as they had significantly more colony-forming units and a higher proliferation index. However, pluripotency and differentiation capacity are more vital for tissue regeneration, and both of these capacities were decreased in PPDLSCs, which expressed stemness-associated genes at lower levels and showed reduced osteogenic and adipogenic differentiation. These results are consistent with a previous report [18] demonstrating that the periodontitic microenvironment can have adverse effects on the characteristics of PPDLSCs and lead to impaired periodontal regeneration.

The purpose of our study was to optimize the function of HPDLSCs and PPDLSCs by modulating their extracellular microenvironment. A previous report showed that a young microenvironment supplied by young PDLSCs can improve the proliferation and differentiation ability of aged PDLSCs [19]. DFCs are young precursor cells existing in the periodontium. Thus, we speculated that DFCs could enhance the function of both HPDLSCs and PPDLSCs by providing a beneficial young microenvironment. Because conventional in vitro culture cannot mimic the complicated environment present during cell growth and development, it is common to use special culture techniques to imitate the in vivo microenvironment and guide cells to a specific differentiation fate [25], [26]. Co-culture methods have thus been developed to promote an optimal cell phenotype by mimicking the natural environment in vivo. Co-culture can also be used to observe interactions between different cell types and explore the mechanisms underlying diseases [27]–[29].

In the present study, we established co-culture systems of DFCs/HPDLSCs and DFCs/PPDLSCs. Oct4, Sox2, and Klf4 are transcription factors associated with self-renewal and pluripotency, and we demonstrated that both HPDLSCs and PPDLSCs showed higher expression of these genes after co-culture with DFCs, indicating that DFCs can improve the comprehensive potency of HPDLSCs and PPDLSCs. In addition, colony-formation and cell cycle analyses further demonstrated that DFCs can provide a favorable microenvironment to optimize the proliferation capacity of HPDLSCs and PPDLSCs.

Soleymaninejadian et al. [30] reported that MSCs secrete a variety of cytokines and factors, including transforming growth factor-β (TGF-β), hepatic growth factor (HGF), prostaglandin E2 (PGE2), IL-10, nitric oxide (NO), indolamine2, 3-dioxygenase (IDO), heme oxygenase-1 (HO-1), and human leukocyte antigen-G (HLA-G). Many of these soluble factors have been shown to maintain the stemness of MSCs and also increase their proliferation ability [31]. DFCs are a type of MSCs present in periodontal tissue. In our co-culture system, DFCs and HPDLSCs or PPDLSCs were separated by a 0.4 µm polycarbonate membrane, allowing the transport of molecular but not cellular components [32]. Therefore, soluble factors secreted by the DFCs likely diffused into the medium to affect the HPDLSCs and PPDLSCs.

In some studies, opposite effects on proliferation and differentiation have been observed, i.e., when stemness was enhanced, proliferation was enhanced but differentiation was inhibited [33]. Such a phenomenon is beneficial for maintaining the pluripotency of MSCs in vitro but is not ideal for directing tissue regeneration. In our study, in addition to improving the proliferation, co-culture also had advantageous effects on differentiation. We found that DFCs improved the in vitro osteogenic capacity, as osteogenic gene and protein activity, ALP activity and mineralized nodule formation were enhanced. The adipogenic capacity was also improved based on the increased PPARγ expression and the formation of lipid droplets. Transplantation of cells into tissues is considered to be an appropriate method for evaluating the function of the cells [34]. The omentum is usually chosen as the implantation site because it provides sufficient space for the transplantation of immunoprotective tissue as well as an adequate blood supply [35]. Upon transplantation, HPDLSCs co-cultured with DFCs grew well and produced root/periodontal ligament-like and periodontal ligament/bone-like tissues. Several studies [36], [37] have shown that after engraftment, MSCs contribute to tissue repair secretion of trophic molecules, including soluble extracellular matrix glycoproteins, cytokines, and growth factors, and through direct cell-to-cell contact. Moreover, as a type of MSC, DFCs are also young precursor cells. Cells with a young phenotype have been confirmed to enhance the proliferation and differentiation ability of PDLSCs by providing a young microenvironment [19]. However, the environment supplied by DFCs is complicated, which suggests that a combination of multiple factors from DFCs may influence the proliferation and differentiation of HPDLSCs and PPDLSCs, subsequently providing better periodontal regeneration in vivo.

In our study, co-culture with DFCs had a greater effect on PPDLSCs than HPDLSCs. Specifically, the stemness-associated gene expression, number of colony-forming units, proliferation index, ALP activity and osteogenic gene expression were all enhanced to a greater degree. Several studies have indicated that, in addition to secreting trophic factors as mentioned above, MSCs also have immunomodulatory and anti-inflammatory properties [36]. MSCs have been shown to modulate the microenvironment of injured tissues and protect damaged tissues by releasing anti-inflammatory molecules [38], [39]. These molecules may not only reduce inflammation, apoptosis and fibrosis in damaged tissues but also enhance tissue regeneration. The PPDLSCs in this study were derived from an inflammatory microenvironment. Epigenetics studies have shown that PPDLSCs may constitutively secrete inflammatory factors, such as TNF-α and IL-1β, in vitro [40]. Thus, PPDLSCs are distinct from HPDLSCs with regard to both the cell source and the microenvironment. For example, inflammatory factors secreted by PPDLSCs may stimulate the immunomodulatory effects of DFCs, causing the DFCs to produce more anti-inflammatory cytokines and trophic factors, thereby enhancing the biological properties of the PPDLSCs. Future studies should further explore the specific mechanism.

## Conclusion

In summary, our data from in vitro and in vivo assays demonstrated a positive role for DFCs, as a type of MSCs and precursor cells from periodontal tissues, in providing a favorable microenvironment for optimizing the self-renewal and multi-differentiation capacity of HPDLSCs. Furthermore, DFCs helped to ameliorate the biological impairment of PPDLSCs caused by inflammation and may ultimately be useful in enhancing periodontal regeneration using PDLSCs.
